# The Emotions

**Published:** 1855-12

**Authors:** 


### THE EMOTIONS.

We believe strongly in the emotions, as an element of health and disease. We have full frequent demonstrations of this, not a hundred miles away from home; for a day or two in the dumps takes wifey's appetite all away, and she gets "heart sick," as she calls it.

Good emotions, improve digestion, while the bad ones impair it; hence many of our articles have a direct bearing on health, although a dozen of them may be read without naming the words 'health,' 'disease,' 'doses,' or 'symptoms.' So, for "once and the last time," as the auctioneer says, we advertise our readers, that we can't be at the trouble to show what connection any particular subject we discuss has with health; they must work out the problem themselves, under the general rule laid down at the top of the first page of each number.

